# Quercetin, a Flavonoid Antioxidant, Ameliorated Procarbazine-Induced Oxidative Damage to Murine Tissues

**DOI:** 10.3390/antiox4020304

**Published:** 2015-04-28

**Authors:** Ebenezer Tunde Olayinka, Ayokanmi Ore, Oluwatobi Adewumi Adeyemo, Olaniyi Solomon Ola, Olaoluwa Oluwaseun Olotu, Roseline Chinonye Echebiri

**Affiliations:** Biochemistry Unit, Department of Chemical Sciences, Ajayi Crowther University, PMB 1066, Oyo, Oyo State 211213, Nigeria; E-Mails: oreayokanmi@gmail.com (A.O.); dewumyt@gmail.com (O.A.A); olaolaniyis@yahoo.com (O.S.O.); olaoluolot@yahoo.co.uk (O.O.O.); echebiriroseline@gmail.com (R.C.E.)

**Keywords:** procarbazine, oxidative stress, quercetin, antioxidant, rat

## Abstract

Procarbazine (PCZ) (indicated in Hodgkin’s disease), is an alkylating agent known to generate free radicals *in vivo,* while Quercetin (QCT) is a flavonoid antioxidant with proven free radical scavenging capacity. This study investigated the protective effects of QCT on PCZ-induced oxidative damage in the rat. Male Wistar rats (160–180 g) were randomized into five groups (*n* = 5/group): I (control), II PCZ-treated (2 mg/kg body weight (bw) for seven days); III pre-treated with QCT (20 mg/kg bw) for seven days, followed by PCZ for seven days; IV co-treated with PCZ and QCT for seven days and V administered QCT alone for seven days. PCZ caused a significant increase in plasma total bilirubin, urea, and creatinine when compared with control (*P* < 0.05)*.* Similarly, plasma activities of alkaline phosphatase (ALP), aspartate aminotransferase (AST), alanine aminotransferase (ALT), and γ-glutamyl transferase (γ-GT) were significantly increased in the PCZ-treated group relative to control. Furthermore, PCZ caused a significant decrease in the activities of hepatic superoxide dismutase (SOD), catalase (CAT) and glutathione-*S*-transferase (GST) as well as levels of ascorbic acid (AA) and glutathione (GSH). This was followed by a significant increase in hepatic malondialdehyde (MDA) content. However, QCT pre-treatment and co-treatment ameliorated the PCZ-induced changes in plasma levels of urea, creatinine, and bilirubin as well as the activities of ALP, AST, ALT, and GGT. QCT also ameliorated hepatic AA and GSH levels and the activities of SOD, CAT, and GST. This all suggests that QCT protected against PCZ-induced oxidative damage in rats.

## 1. Introduction

**Figure 1:**
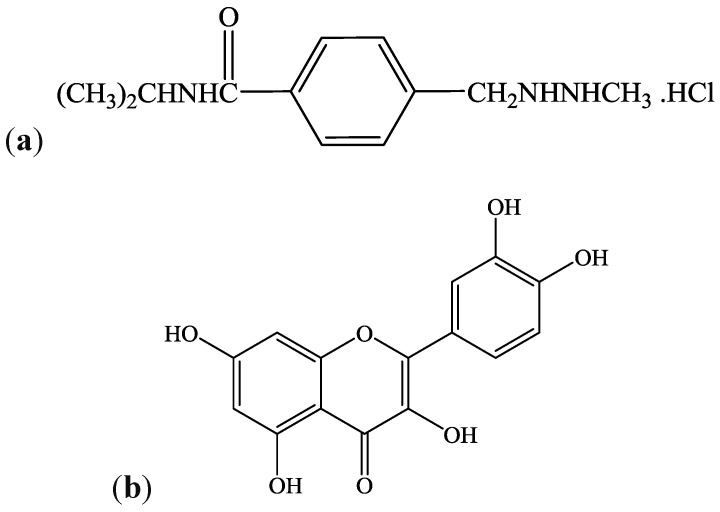
Chemical structures of Procarbazine (*N*-isopropyl-a-(2-methyl-hydrazine)-*p*-toluamide hydrochloride) (**a**), and Quercetin (3,5,7,3′,4′-pentahydroxyflavone) (**b**).

Procarbazine (*N*-isopropyl-a-(2-methyl-hydrazine)-*p*-toluamide hydrochloride) (PCZ) is an orally administered alkylating anticancer agent used for the treatment of Hodgkin’s lymphoma, malignant melanoma, and brain tumors in children (Figure 1a). It is also administered as a component of a chemotherapeutic cocktail used in the treatment of Hodgkin’s disease, melanoma, and bronchogenic carcinoma [1]. PCZ is a prodrug, and therefore requires extensive metabolism for its bioactivation to reactive intermediates—a process involving CYP2B6 and CYP1A enzymes [2]. Following oral administration, it is first oxidized to azoprocarbazine and further oxidized to a mixture of methylazoxyprocarbazine and benzylazoxyprocarbazine isomers [3]. The methylazoxyprocarbazine produced has been proposed as the active metabolite responsible for anticancer activity in leukaemia [4].

The oxidation of PCZ by microsomal P450 systems and peroxidases produce free radical species. Active methyl and benzyl radicals have been shown to be formed through a nitrogen-centered radical intermediate following one-electron oxidation of PCZ [5,6]. The nitrogen-centered radical is postulated to undergo rearrangements to form carbon-centered radicals and nitrogen [6,7]. Although free radical formation and oxidative stress have been proposed as part of the pharmacological mechanisms of most alkylating agents [8], they also appear to contribute to the organ toxicities exhibited by these drugs [9]. Moreover, an excessive physiological level of free radicals, if not neutralized will lead to cell and tissue damage [8,10]. Some of the reported toxicities associated with procarbazine include mutagenicity [11], nephrotoxicity [12], granulomatous hepatitis [13], hepatotoxicity [14], as well as testicular and spermatotoxicity [15,16].

An array of antioxidant defense mechanisms present in the cell play a vital role in maintaining the physiological redox status and protect against the harmful effect of toxic drug metabolites and free radicals. These include the non-enzymic antioxidants like reduced glutathione (GSH), ascorbic acid (AA), and vitamin E among others and the enzymic antioxidants such as glutathione-*S*-transferase (GST), glutathione peroxidase (GPx), glutathione reductase (GR), superoxide dismutase (SOD), and catalase (CAT) [17]. Quercetin (QCT), Figure 1b, is a flavonoid antioxidant (of the flavonol subclass) which is ubiquitous in plants and plant food sources [18]. QCT and its derivatives have been promoted in several studies as potent antioxidants [15,19]. In addition, QCT possesses a number of pharmacological activities including antioxidant, anticancer, antimicrobial, and antiviral [20,21]. In recent studies, QCT was reported to protect against drug-induced genotoxicity [22], hepatotoxicity [23], nephrotoxicity [15], lung injury [24], and oxidative stress *in vivo* [25].

One of the challenges associated with the use of alkylating agents is the phenomenon of drug-induced toxicity. Considering the popular use of alkylating agents in chemotherapy, it is thought that antioxidant supplementation may offer protection against toxic side effects. Use of antioxidants along with anticancer agents has been demonstrated to have little or no effect on anticancer activity [26]. For instance, QCT used in the present study was reported to protect against anticancer drug-induced toxicity *in vivo* without compromising antitumor activity [27]. Rather, it is known to improve the chemotherapeutic efficacy of certain alkylating agents *in vivo* [28]. Moreover, comparative studies in rats and in Hodgkin’s lymphoma patients suggest that human biotransformation of procarbazine is similar to that of rat [29]. Little or no work on the protective effect of QCT on PCZ-induced oxidative damage has been reported previously. Consequently, the present study was designed to investigate the protective effect of quercetin pre-treatment and co-treatment on procarbazine-induced nephrotoxicity, hepatoxicity, and oxidative stress in the rat model.

## 2. Materials and Methods

### 2.1. Chemicals and Reagents 

The following substances were employed: Procarbazine Hydrochloride Capsules (Naprod Life Sciences Pvt. Ltd., Mumbai, India); Quercetin, Glutathione, 1-chloro-2,4-dinitrobenzene (CDNB), 5,5′-dithio bis-2-nitrobenzoic acid (DTNB), epinephrine, and hydrogen peroxide (H_2_O_2_), (Sigma^®^ Chemical Company, London, UK); Assay kits for alanine aminotransferase (ALT), aspartate aminotransferase (AST), alkaline phosphatase (ALP), gamma glutamyl transferase (GGT), urea, Creatinine, total bilirubin (RANDOX^®^ Laboratories Ltd., Antrim, UK). All other reagents used in the study were of analytical grade and highest purity.

### 2.2. Animal Selection and Care

Twenty five male Wistar rats weighing between 160 and 180 g were obtained from the animal holding unit, Department of Chemical Sciences, Ajayi Crowther University, Oyo, Nigeria. The rats were acclimatized under laboratory conditions prior to experiment. The animals were housed in wire-meshed cages and provided with food and water *ad libitum*. The animals were maintained at standard conditions of temperature and humidity with 12 h light/dark cycles. They were fed with commercial rat diet (Ladokun^®^ Feeds, Nigeria Ltd., Ibadan, Nigeria). The study was approved by the ethical committee of the Faculty of Natural Sciences, Ajayi Crowther University, Oyo, Nigeria. Handling of the experimental animals also conforms to international guidelines on the care and use of laboratory animals (National Research Council) [30].

### 2.3. Animal Grouping and Drug Treatments

The animals were randomly assigned into five experimental groups (I–V) of five animals each. The animals of each group were treated as presented in Table 1. The dose for PCZ (2 mg/kg bw) was selected based on the recommended adult dose for Hodgkin’s disease while the dose for QCT (20 mg/kg bw) was arrived at based on available literature [31]. The respective doses were worked out according to the average weight of animals in each treatment groups and delivered in one mL of distilled water. The drug doses were administered once daily by oral intubation.

**Table 1:** Experimental design.

Treatment—Groups	Treatments—Duration	
	Day 1–7	Day 8–14
I (CTRL)	-	Control; distilled water
II (PCZ)	-	2 mg/kg bw PCZ
III (PCZ + QCT-P)	20 mg/kg bw QCT	2 mg/kg bw PCZ
IV (PCZ + QCT-C)	-	2 mg/kg bw PCZ + 20 mg/kg bw QCT
V (QCT-A)	-	20 mg/kg bw QCT

### 2.4. Animal Sacrifice, Collection of Blood and Liver Samples

Blood samples were collected from each animal 24 h after the final treatments, through retro orbitals plexus into heparinized tubes (Li heparin). Animals were thereafter sacrificed and liver was carefully excised from each animal for preparation of the cytosolic fraction.

### 2.5. Preparation of Plasma and Cytosolic Fractions

Plasma was obtained by centrifugation of whole blood sample at 4000 rpm for 5 minutes using a CENCOM^®^ bench centrifuge (Analytika, Athens, Greece). The plasma obtained were stored at –4 °C for subsequent plasma assays. The liver excised from each rat was blotted of blood stains, rinsed in ice-cold 1.15% KCl and homogenized in 4 volumes of ice-cold 0.01 M potassium phosphate buffer, (pH 7.4). The homogenates were centrifuged at 12,500× *g* for 15 min at –4 °C (Eppendorf UK Ltd., Stevenage, UK) and the supernatants, termed the post-mitochondrial fractions (PMF) were aliquoted and used for subsequent biochemical assays.

### 2.6. Determination of Plasma and Liver Protein Content

The protein concentration in the plasma and liver PMF was determined according to the biuret method of Gornall *et al.* [32], based on the reaction of peptide bonds in proteins with Cu^2+^ in moderately alkaline medium. This results in a purple colored chelate of the protein with maximum absorbance at 540 nm. The intensity of the purple color is proportional to the amount of protein present. The reaction mixture consists of 4 mL of biuret reagent and 1 mL of appropriately diluted sample. A blank was prepared with 4 mL of biuret solution and 1 mL of distilled water. The protein concentration in the samples was extrapolated from the standard bovine serum albumin (BSA) curve.

### 2.7. Assay of Biomarkers of Nephrotoxicity

Plasma urea and creatinine was determined with RANDOX^®^ diagnostic kits following the manufacturer’s protocol. The method for creatinine assays was based on a colorimetric alkaline picrate method [33] with creatinine-picrate complex measured at 492 nm. Plasma urea determination was based on the Fenton reaction [34] with the diazine chromogen formed absorbing strongly at 540 nm.

### 2.8. Assay of Biomarkers of Hepatotoxicity 

Biomarkers of hepatotoxicity, plasma total bilirubin (TBILI) level, and activities of alkaline phosphatase (ALP), alanine aminotransferase (ALT), aspartate aminotransferase (AST), and gamma glutamyl transferase (γ-GT) were assayed using RANDOX^®^ diagnostic kits based on the manufacturer’s procedure. Assay of TBILI level was based on the dimethyl sulphoxide method of Tietz *et al*. [35]. The dimethyl sulphoxide formed a colored compound with maximum absorption at 550 nm. ALP activity was determined in accordance with the principles of Tietz [35]. The *p*-nitrophenol formed by the hydrolysis of *p*-nitrophenyl phosphate confers a yellowish color to the reaction mixture and its intensity can be monitored at 405 nm to give a measure of enzyme activity. Determination of plasma ALT and AST activities was based on the principle described by Reltman and Frankel [36]. ALT activity was measured by monitoring the concentration of pyruvate hydrazone formed with 2,4-dinitrophenylhydrazine at 546 nm. AST activity was measured by monitoring the concentration of oxaloacetate hydrazone formed with 2,4-dinitrophenylhydrazine at 546 nm. γ-GT activity was determined following the principle described by Szasz [37]. The substrate L-γ**-**glutamyl-3-carboxy-4-nitroanilide, in the presence of glycylglycine is converted to 5-amino-2-nitrobenzoate by γ-GT measured at 405 nm. The increase in absorbance is proportional to γ-GT activity.

### 2.9. Assay for Non-Enzymatic Antioxidants in the Liver

#### 2.9.1. Hepatic Reduced Glutathione Level (GSH)

The level of hepatic GSH was determined according to the method of Jollow *et al*. [38]. The chromophoric product resulting from the reaction of Ellman’s reagent (5,5′-dithiobis-(2-nitrobenzoic acid), DTNB) with the reduced glutathione, 2-nitro-5-thiobenzoic acid possesses a molar absorption at 412 nm which was read using a spectrophotometer. Briefly, the reaction mixture was made up of 0.2 mL of sample, 1.8 mL of distilled water and 3 mL of 4% sulphosalicylic acid. The mixture was allowed to stand for 5 minutes and then filtered. 1 mL of the filtrate was added to 4 mL of 0.1 M phosphate buffer and finally, 0.5 mL of Ellmans’ reagent (0.04% in 0.1M phosphate buffer, pH 7.4) was added. A blank was prepared with 4 mL of the 0.1 M phosphate buffer, 1 mL of diluted sulphosalicylic acid and 0.5 mL of the Ellman’s reagent. The absorbance was measured at 412 nm. GSH concentration in the samples was estimated from the standard curve for GSH.

#### 2.9.2. Hepatic Ascorbic Acid Level (AA)

The ascorbic acid (AA) concentration in the liver PMF was determined according to the method of Jagota and Dani [39]. In this procedure, AA in biological samples reacts with Folin Ciocalteu (Folin-phenol) reagent, an oxidizing agent to give a blue color which has maximum absorbance at 760 nm. Only strong reductants like ascorbic acid can react with Folin-phenol reagent under acidic conditions and interference by other possible substances is eliminated. Briefly, 0.5 mL of test sample was added to 0.8 mL of 10% TCA in a test tube. After vigorous shaking, the tubes were kept in an ice bath for 5 minutes and centrifuged at 3000× *g* for another 5 min. Two mL of supernatants were added to 0.2 mL of Folin’s reagent (diluted 10 fold in ddH_2_O) and stirred vigorously. After 10 min, the absorbance of the blue color developed was measured using a spectrophotometer at 760 nm. The ascorbic acid concentration (μg/mL) in the liver post mitochondrial fraction was extrapolated from the ascorbic acid standard curve. A standard curve was prepared by taking varying concentrations of standard ascorbic acid in ddH_2_O, ranging from 0.05 to 0.7 mL using a procedure similar to the one above.

### 2.10. Assay of Hepatic Antioxidant Enzymes

#### 2.10.1. Hepatic Glutathione *S*-Transferase (GST) Activity

Hepatic GST activity was determined by the method described by Habig *et al*. [40] using 1-chloro-2,4-dinitrobenzene (CDNB) as substrate. Briefly, the assay mixture (3 mL) was made up of 30 μL of reduced GSH (0.1 M), 150 μL of CDNB (3.37 mg/ mL), 2.79 mL phosphate buffer (0.1 M, pH 6.5) and 30 μL of liver PMF. The reaction was allowed to run for 60 seconds before the absorbance was measured at 340 nm against the blank. GST activity in the liver homogenate was determined from the following equation:

$$GST\;Activity\;\left(\mu mole/\;min/mg\;protein\right)=\frac{Abs/min}{9.6}\times \frac{1}{0.03\times protein\;(mg)}$$

9.6, the molar extinction coefficient of CDNB (mmol^−1^cm^−1^); 0.03, volume of PMF used in mL.

#### 2.10.2. Hepatic Superoxide Dismutase (SOD) Activity

The procedure of Misra and Fridovich [41] was used for the determination of hepatic superoxide dismutase (SOD) activity by measuring the inhibition of auto-oxidation of epinephrine at pH 10.2. One mL of the sample was diluted in 9 mL of distilled water to obtain a 1 in 10 dilution. An aliquot of 0.2 mL of the diluted enzyme preparation was added to 2.5 mL of 0.05 M carbonate buffer (pH 10.2), equilibrated in the spectrophotometer, and the reaction was started by the addition of 0.3 mL of freshly prepared 0.3 mM epinephrine to the mixture which was quickly mixed by inversion. The reference cuvette contained 2.5 mL of carbonate buffer, 0.3 mL of adrenaline and 0.2 mL of distilled water. The increase in absorbance at 480 nm was monitored every 30 s for 150 s. Activity of SOD in the liver PMF was expressed as follows:

$$\%\;Inhibition=\frac{Increase\;in\;Abs\;of\;sample}{Increase\;in\;Abs\;of\;blank}\times 100$$

One Unit of SOD activity is defined as the amount of SOD necessary to cause 50% inhibition of the oxidation of adrenaline to adrenochrome over an interval of one minute.

$$SOD\;activity\;(Unit/mg)=\frac{SOD(Unit)}{Total\;protein\;(mg)}\times df$$

d*f* = dilution factor

#### 2.10.3. Hepatic Catalase Activity

Hepatic catalase activity was determined by the method described by Singha [42] based on the reduction of dichromate in acetic acid to chromic acetate when heated in the presence of hydrogen peroxide (H_2_O_2_). The assay mixture, 4 mL of H_2_O_2_ solution (800 μmoles), 5 mL of phosphate buffer (0.01 M, pH 7.0), 1 mL of diluted liver PMF (1:50) was rapidly mixed at room temperature. A 1 mL portion of reaction mixture was withdrawn and blown into 2 mL dichromate/acetic acid reagent at 60 seconds intervals to determine the amount of H_2_O_2_ remaining. The chromic acetate produced was measured spectrophotometrically at 570 nm and the amount of H_2_O_2_ remaining was extrapolated from the standard curve for H_2_O_2_. Catalase activity was expressed as micromole of H_2_O_2_ consumed per min per mg protein.

### 2.11. Assay of Hepatic Level of Lipid Peroxidation

The extent of lipid peroxidation (LPO) in the liver was estimated by the method of Vashney and Kale [43]. The method involved the reaction between malondialdehyde (MDA; product of lipid peroxidation) and thiobarbituric acid to yield a stable pink chromophore with maximum absorption at 532 nm. Briefly, the reaction mixture consisted of 1.6 mL Tris-KCl buffer, 0.4 mL of the test sample, 0.5 mL of 30% TCA, 0.5 mL of 0.75% TBA and the mixture was placed in a water bath for 1 h at 95 °C. This was then cooled in ice and centrifuged at 3000 rpm. The clear supernatant was collected and the absorbance measured against a reference blank of distilled water at 532 nm in a spectrophotometer. Lipid peroxidation in nmole/mg protein was computed as:

$$MDA=\frac{Abs\times Volume\;of\;Reaction\;Mixture}{E_{532}\times Volume\;of\;Sample\times mg\;protein}$$

where *E*_532_ is the molar extinction coefficient for MDA = 1.56 × 10^5^ M^−1^ Cm^−1^

### 2.12. Statistical Analysis

Data are presented as the mean ± standard deviation (SD) of five replicates. Statistical significance was determined by one-way analysis of variance (ANOVA) followed by Duncan’s multiple comparison between control and treated rats in all groups using SigmaPlot^®^ statistical package (Systat Software Inc., San Jose, CA, USA). *P*-values less than *0.05* (*P* < 0.05) were considered statistically significant.

## 3. Results

### 3.1. QCT Pre-Treatment and Co-Treatment Protects against PCZ-Induced Nephrotoxicity in Rats

**Figure 2:**
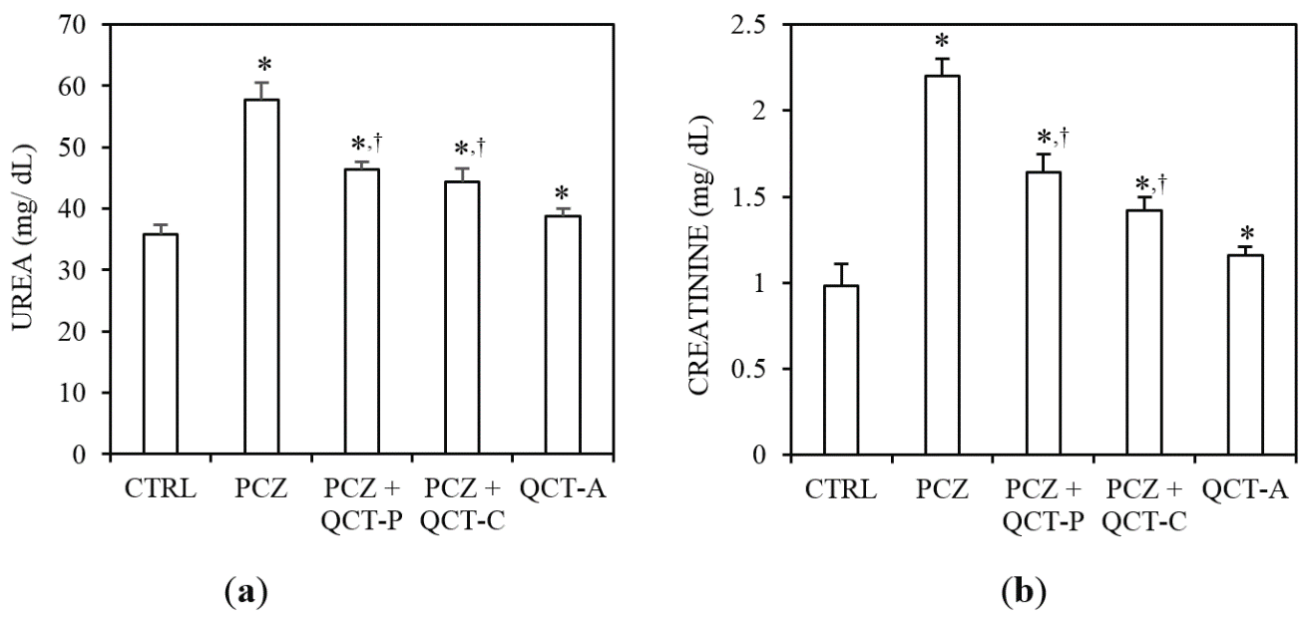
Protective effect of QCT pre-treatment and co-treatment against PCZ-induced changes in biomarkers of nephrotoxicity in rats. Data represent the means ± SD for five rats in each group; * significantly different from the CTRL; ^†^ significantly different from PCZ (*P* < 0.05).

Figure 2 represent the protective effect of QCT pre-treatment and co-treatment against PCZ-induced changes in plasma urea and bilirubin levels in rat. Administration of PCZ caused a significant (*P* < 0.05) increase in plasma urea level by 60.9% compared to control (Figure 2a). In a similar manner, plasma creatinine (Figure 2b) increased significantly by 124.5% relative to the control (*P* < 0.05). However, pre-administration and co-administration of QCT with PCZ attenuated the observed elevated plasma urea and creatinine levels when compared with the PCZ-treated group.

### 3.2. QCT Pre-Treatment and Co-Treatment Protects against PCZ-Induced Hepatotoxicity in Rats

**Figure 3:**
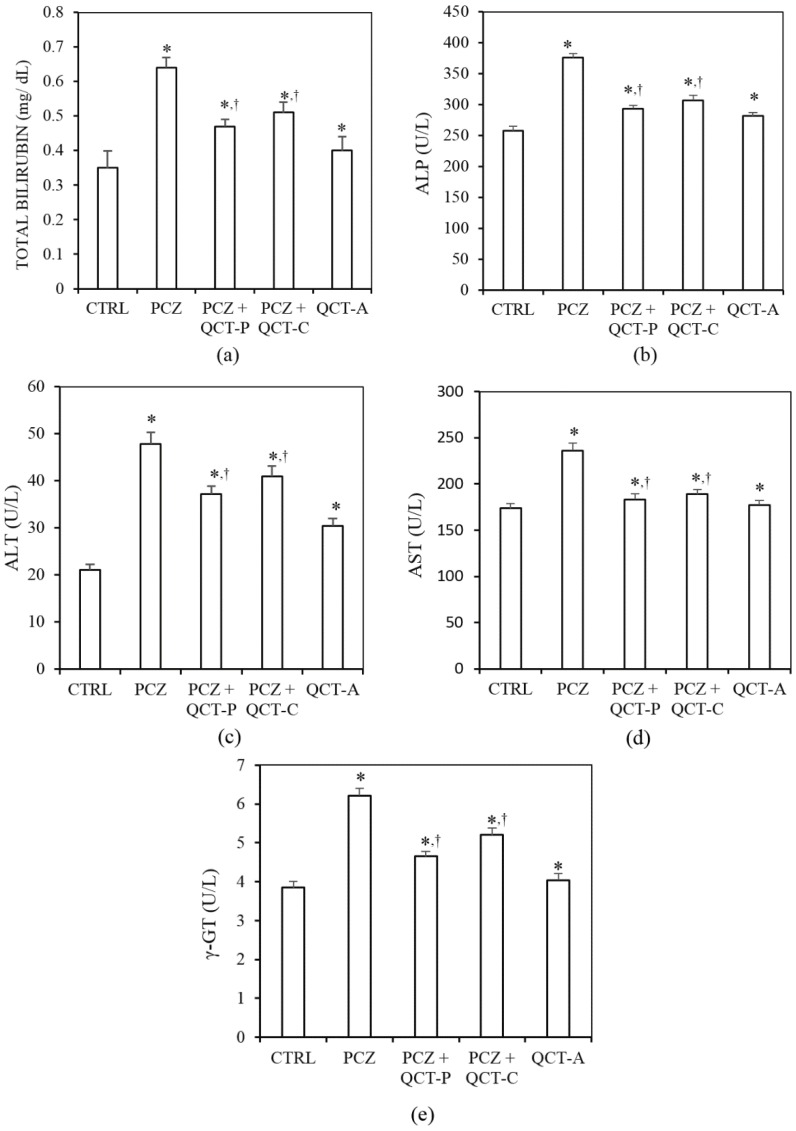
Protective effect of QCT pre-treatment and co-treatment against PCZ-induced changes in biomarkers of hepatotoxicity in rats. Data represent the means ± SD for five rats in each group; * significantly different from the CTRL; ^†^ significantly different from PCZ (*P* < 0.05)*.*

The influence of QCT pre-treatment and co-treatment on PCZ-induced changes in biomarkers of hepatotoxicity in rats is presented in Figure 3. The plasma level of total bilirubin (TBILI) and alkaline phosphatase (ALP) activity (which are biomarkers of hepatobiliary damage) increased significantly (*P* < 0.05) in rats by 83.3% and 46.7% respectively following PCZ treatment (Figure 3a,b). However, the levels of TBILI and ALP activity were significantly ameliorated in the plasma of animals pre-treated or co-treated with QCT when compared with the PCZ group. 

PCZ treatment also caused a significant increase in the activities of alanine aminotransferase (ALT), aspartate aminotransferase (AST), and gamma glutamyl transferase (γ-GT) (biomarkers of hepatocellular toxicity) in the plasma of rats by 127%, 36% and 62% respectively compared to values in control (Figure 3c–e). Pre-treatment and co-treatment of QCT with PCZ significantly ameliorated the elevated activities of plasma ALT, AST, and γ-GT when compared to PCZ-treated group.

### 3.3. QCT Pre-Treatment and Co-Treatment Protects against PCZ-Induced Oxidative Stress in the Liver of Rats

**Figure 4:**
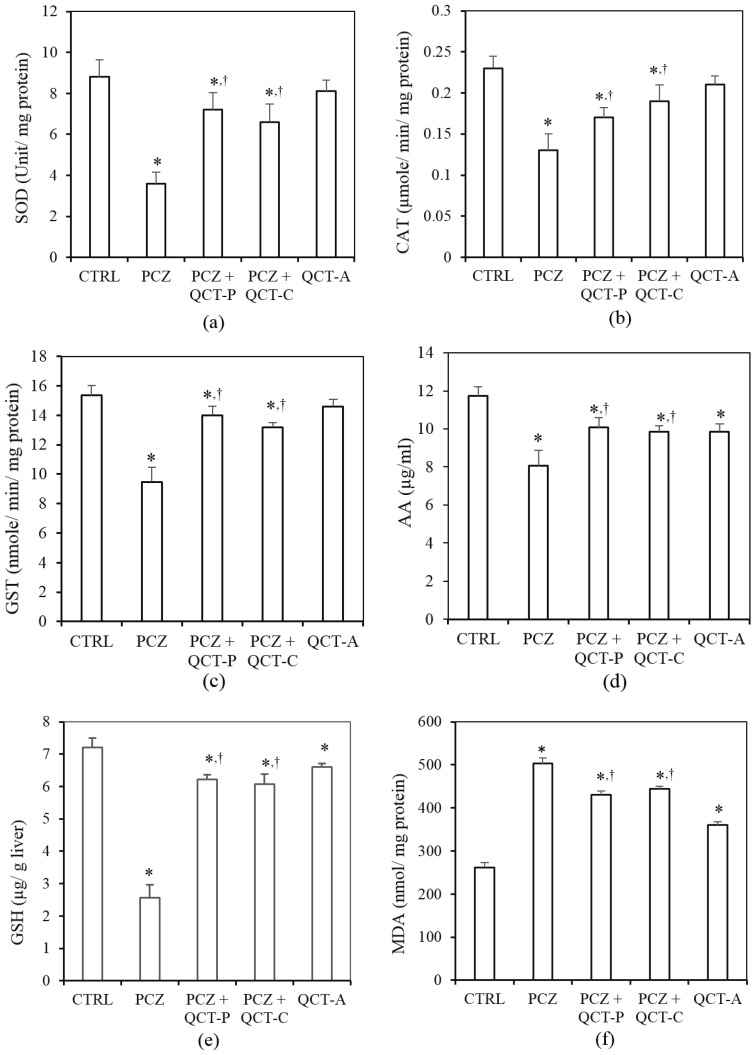
Protective effect of QCT pre-treatment and co-treatment against PCZ-induced changes in hepatic biomarkers of oxidative stress in rats. Data represent the means ± SD for five rats in each group; * significantly different from the CTRL; ^†^ significantly different from PCZ (*P* < 0.05).

The protective effect of QCT pre-treatment and co-treatment against PCZ-induced changes in hepatic biomarkers of oxidative stress in rats is presented in Figure 4. Hepatic SOD activity (Figure 4a) was significantly reduced in the PCZ group by 59.1% when compared with control (*P* < 0.05). Similarly, hepatic CAT activity (Figure 4b) was significantly decreased following PCZ-treatment, by 43.5% when compared with control. Hepatic GST activity (Figure 4c) was also significantly reduced by 38.3% in the PCZ-treated rats when compared with the control. However, QCT pre-treatment and co-treatment significantly ameliorated the PCZ-induced decrease in hepatic activities of SOD, CAT, and GST when compared with the PCZ group (*P* < 0.05). 

The influence of PCZ and QCT pre- and co-treatment on hepatic levels of the non-enzymatic antioxidants, AA and GSH is shown in Figure 4d,e respectively. Following treatment with PCZ, the hepatic AA level was significantly (*P* < 0.05) decreased by 31.2% when compared with the control. Furthermore, PCZ caused a significant depletion of hepatic GSH by 64.3% when compared with the control. Conversely, pre-treatment and co-treatment with QCT significantly (*P* < 0.05) protects against the PCZ-induced decrease in hepatic AA and GSH levels when compared with the PCZ group.

Figure 4f shows the protective effect of QCT on PCZ-induced changes in hepatic malondialdehyde (MDA) level in rats. The hepatic MDA content rose significantly (*P* < 0.05) in the PCZ-treated rats by 90.8% when compared with the control. However, QCT pre-treatment and co-treatment attenuated the increase in hepatic MDA when compared with the PCZ-treated group.

## 4. Discussion

Drug-induced oxidative stress is a relevant side effect of most anticancer drugs, especially of alkylating agents. That is why the present study focused on the ameliorative potential of quercetin (QCT), against procarbazine (PCZ)-induced oxidative damage in rats. In our previous study, we observed the potential nephrotoxic, hepatotoxic, and pro-oxidant effect of an alkylating agent in the rat model [15]. Several studies have promoted QCT and its analogues as excellent antioxidants *in vivo* [21,25,44]. Separately from its antioxidant capacity, QCT also possesses antitumor activity against different cancer cell types [45,46,47]. While information regarding the potential interaction of QCT with PCZ is still lacking, data from previous studies suggest that QCT improved chemotherapeutic efficacy of most anticancer agents [48,49,50]. In a more recent study [51], QCT was found to enhance the antitumor activities of certain topoisomerase inhibitors.

To establish the protective effect of QCT in PCZ-induced toxicity, biomarkers of nephrotoxicity, hepatotoxicity, and oxidative stress were assessed. Plasma urea and creatinine levels are established biomarkers of nephrotoxicity in human and animal subjects [52,53]. Urea and creatinine are metabolic products filtered freely from circulation by the kidney and an increase in plasma level of these substances is an indication of alteration in renal function [54]. Data from this study suggest that PCZ alters renal function leading to accumulation of these substances in the plasma of rats. The observed increase in plasma urea and creatinine in this study is consistent with a previous report on alkylating drugs [25]. Our results indicate that the natural flavonoid QCT protects the kidneys from the toxic damage caused by PCZ. QCT was reported previously as a potent nephroprotective agent in anticancer drug-induced nephrotoxicity [55,56].

The liver is involved in the metabolic transformation of drugs, which predisposes it to drug-induced damages. Plasma levels of TBILI as well as activities of the liver enzymes, ALT, AST, ALP, and γ-GT, are reliable markers of hepatotoxicity [57]. Bilirubin is present in the liver, bile, intestines, and the reticuloendothelial cells of the spleen while ALP and γ-GT are associated with the cell membrane [57]. Increase in plasma TBILI, ALP, and γ-GT is known to be associated with impairment of intrahepatic and extrahepatic bile flow (cholestasis), hepatobiliary injury, erythrocyte destruction or altered bilirubin metabolism [58,59]. The observed increase in plasma TBILI, ALP, and γ-GT is consistent with previous reports [9,60]. Plasma activities of ALT and AST are well established biomarkers of hepatocellular integrity *in vivo* [61]. Increase in the activities of ALT and AST in the plasma may have resulted from leakage from damaged hepatocytes [61]. In this study, pre- and co-treatment with QCT ameliorated the PCZ induced hepatic injuries in rats. Our findings also agree with previous findings showing the hepatoprotective activity of QCT [9,23,62].

Previous studies established a link between hepatotoxicity and oxidative stress [63,64], occasioning the consideration of the effect of PCZ on biomarkers of oxidative stress *in vivo*. Alkylating agents including PCZ are known to be associated with production of reactive oxygen species (ROS) leading to depletion of cellular detoxifying thiols and antioxidant enzymes [14,15]. In this study, PCZ caused a significant decrease in the activities of hepatic SOD, CAT, and GST. SOD catalyzes the rapid dismutation of superoxide radical to hydrogen peroxide and molecular oxygen while CAT converts the hydrogen peroxide formed in this process and other cellular processes into water and molecular oxygen [65]. GST on the other hand is involved primarily in the detoxification of xenobiotics in the liver [66] and it also forms a vital component of the antioxidant defense system [67]. The low molecular weight antioxidant molecules, AA and GSH, play a crucial role in cellular redox balance. Both AA and GSH are involved in scavenging free radicals in cells and are often the first line of defense against oxidation [68]. AA functions in the aqueous phase and is involved in the preservation of tocopherol in membranes and lipoproteins [69]. GSH acts as a cofactor for several enzymic antioxidants like glutathione peroxidase (GPx), glutathione-*S*-transferase, and is also involved in free radical scavenging activities in the cell [70]. The observed decrease in levels of these antioxidants caused by PCZ may predispose the liver to oxidative damage. This may also be connected to the observed elevation of liver enzymes in the plasma of animals in the PCZ group. In this study, QCT significantly improved the redox balance in the liver of rats which also corroborates earlier findings [15,71]. QCT and its metabolites possess the capacity to neutralize the free radicals from PCZ by donating electrons which can end the electron chain reactions [72]. QCT is one of the most abundant flavonoids in the human diet and is reported to exhibit a wide range of pharmacological activities [21,72], including hepatoprotection observed in the present study.

The phenomenon of lipid peroxidation (LPO) is a physiological event that occurs in normal cells to some extent. LPO is a well-established mechanism of cellular injury in animals, and is used as a marker of oxidative stress and tissue damage *in vivo* [73,74]. An increase in LPO (as shown by an increase in MDA level) was recorded following administration of PCZ. LPO is normally initiated by the attack of free radicals on unsaturated fatty acids [74] leading to the formation of a complex series of compounds including MDA, lipid peroxides, and reactive carbonyl compounds [75]. The fluid properties in biological membranes are due to the presence of polyunsaturated fatty acids (PUFAs) in phospholipids molecules located in both sites of the lipid bilayer [76]. Excessive membrane lipid peroxidation may result in cell disruption. The amelioration of hepatic LPO by QCT in the present study may be attributed to the quenching of free radicals, lipid peroxides, and promotion of antioxidants in the hepatocytes [77,78,79]. Flavonoids including QCT have been reported to interact with membrane lipid components, with a resultant protection of the membranes against oxidative damage [15,80,81,82], thereby preserving the normal cell functions. 

## 5. Conclusions

In the present study, we demonstrated that QCT possesses the capacity to protect against PCZ-induced oxidative damage in rat tissues. This observation suggests a possible use of QCT as a supplement during a prolonged period of chemotherapy with drugs in the categories of PCZ.

## Abbreviations

PCZ: Procarbazine (2 mg/ kg bw)
bw: Body weight
CTRL: Control
QCT: Quercetin
QCT-P: Quercetin pre-treated
QCT-C: Quercetin co-treated
QCT-A: Quercetin-alone
PMF: Post Mitochondrial Fraction
BSA: Bovine Serum Albumin
ALP: Alkaline phosphatase
ALT: Alanine aminotransferase
AST: Aspartate aminotransferase
γ-GT: Gamma glutamyl transferase
SOD: Superoxide dismutase
CAT: Catalase
GST: Glutathione-*S*-transferase
AA: Ascorbic acid
GSH: Reduced Glutathione
LPO: Lipid peroxidation
MDA: Malondialdehyde
ROS: reactive oxygen species
H_2_O_2_: hydrogen peroxide
